# Abstract of Professor Mitchill's Lectures on the Origin and Constitution of the Atmosphere, as Delivered in the New Institution at New York: Being a Section of His Course of Natural History

**Published:** 1809-09

**Authors:** 


Medical and Phyfical Journal.

VOL. XXII.] SEPTEMBER, 1809. [NO. 127.

*Printed for R. PHILLIPS, by W. Thorne, Red Lion Court, Fleet Street, London.*

### *Abstract of* PROFESSOR MITCHILL'S *Lectures on the Origin and Constitution of the Atmosphere, as delivered in the New Institution at New York: being a Section of his Course of Natural History.*

THIS Course is divided by Professor Mitchill into the following Sections: I. GEOLOGY, or the History of the Earth. II. PHOTOLOGY, or the History of Light. III. PYROLOGY, or the History of Heat. IV. HYDROLOGY, or the History of Water. V. ÆROLOGY, or the History of the Atmosphere. VI. MINERALOGY, or the History of the solid Materials of the Globe. VII. BOTANY, or the History of Vegetables. VIII. ZOOLOGY, or the History of Animals. IX. URANOLOGY, or the History of the Solar and Sidereal Systems, the Zodiac, and the other Constellations.

The view he took of the atmosphere, was founded on Lavoisier's declaration that it was formed by the conversion of different substances to gases by caloric. Four substances commonly present: 1. Azotic gas. 2. Oxygenous gas. 3. Carbonic acid gas; and 4. Inflammable gas. The *metallic* constitution of the atmosphere is stated and maintained by him. The late experiments on the alkalies countenance the idea, that azotic is a *metallic gas*, as quicksilver is a *metallic liquid*. It probably rose first from chaos, and is the oldest, as well as most abundant ingredient of the atmosphere. Its natural and chemical qualities were then explained. 2. Evolution of oxygenous air from water, by sharp points and sun-shine, as shown by Rumford's decomposition of water by metals, attracting its phlogiston (or hydrogen) to themselves, and discharging the oxygen in union with light and caloric into the atmosphere. The atmosphere enlarged, and the quantities of water diminished thereby. Origin of plants, aquatic and terrestrial, and their agency in decompounding water, and replenishing the air with oxygen. Thus the atmosphere, by degrees, became respirable, and fit for the economy and life of animals. Eulogy on the Oxonian, Mayow, and an account of his brilliant discoveries, in an age not prepared to comprehend them. 3. As combustion and respiration could now go on, carbonic acid would be readily formed, and was, in all probability, the third ingredient of the atmosphere. History of carbon, carbonic oxyd, carbonic acid, and carbonic acid gas. 4. Phlogistous gas, the fourth constituent part of the atmosphere. Its history and relations. The strong attraction of its radical hydrogen for metals, sulphur, phosphorus, carbon, and inflammable bodies generally. The material of blaze or flame. Explanation of the thermo-lamp, or gas lights, which are nothing more than the inflammable gas that forms the blaze, burning at a distance from the fire-brand and ignited coal which affords it, instead of burning in contact with them, or near their surface. Its extraordinary lightness, capacitates it to raise balloons. The history of these heavy bodies elevated by inflammable air. Proportions, of azote, 783; oxygen, 210; carbonic acid gas, 4; and of phlogistic gas, 3, to constitute 1,000 parts of atmosphere; though different experimenters differ somewhat in the respective quantities of each.

Other ingredients in the atmosphere; vast quantity of water; needful to be guarded against in nice experiments, and making a considerable proportion of its ponderable mass. Water, when elevated, carries with it saline and earthy matters. Examples from the saltness of the air, in maritime situations, made evident to the senses and proved by chemical tests; also demonstrated over and again by Marcgraff, Boyle, Boerhaave, and Linne. Hence, a portion of the earth dissolved in water rises with it, and may be explained without the hypothesis of conversion or transmutation.

Inflammable or phlogistous air is singularly constituted, from its numerous relations and affinities to elevate substances into the higher regions of air. Its attraction for sulphur, phosphorus and carbon, not confined to the solid states of each of them. When the phlogiston was relatively small, and the other ingredients large, they chain the hydrogen to the earth, and the union of the two forms common firm brimstone, coal and phosphorus. But when the quantity of hydrogen is relatively large, and the other materials small, it breaks loose from its confinement, and carries aloft with it a portion of the several ingredients with which it is combined, and raises them as high high as it mounts. So it may dissolve and elevate the metals, and especially iron, to which it has a readiness for association. When the metal is relatively inconsiderable and the phlogistous gas copious, the heavier body may be elevated by the lighter to a very great height. On the contrary, when the metallic ingredient is large, and the phlogistic small, the former represses the latter, and overcomes its tendency upwards. Whenever, therefore, any of the before mentioned substances are combined with inflammable air in due quantities, they may ascend with it into the upper spaces of the atmosphere, even though they are metals.

On considering this body of evidence, Prof. Mitchill drew another argument in favor of the elevation of earthy and metallic substances, from the fall of stones from the atmosphere. If it could be comprehended how the constituent parts of these solid bodies could be carried to great heights in the atmosphere, there might be a possibility of conceiving how such meteoric masses could be formed. Generally, these atmospherical concretions are accompanied by flame and light. The bodies formed are a composition of earthy and metallic ingredients. They carry with them the signs of scorching and fusion. There are no materials found in them different from those which our planet affords. But they are connected or associated in an assemblage that differs from any mineral hitherto discovered on our globe. Now, all these circumstances are natural and obvious enough; for it is probable that the blaze of the meteor was inflammable air. This probably held metallic particles in solution. On the burning of this gas, those particles would be discharged from their connection, and precipitated. But in order to make this inflammable gas explode, the concurrence of oxygenous air would be necessary; and the consumption of this would dislodge a considerable quantity of the earthy matters it held in solution. These would be mingled at the moment of discharge with the metallic particles. The heat of such a combination of burning gases would be intense. The earthy and metallic particles would be likely to combine, by virtue of cohesive and chemical attractions. Their union would be promoted by the actual heat, and wafting and melting might naturally enough be the consequence of its intenseness. As their association is formed under circumstances which differ from those which obtain on the surface of the earth, so the mixture and proportions of the ingredients also differ; and the meteoric stones, containing no new elements but merely new mixtures of those already known, are such combinations of matter, as, according to correct theory, we should suppose they ought to be.

Contenting himself with this survey of the subject, Professor Mitchill said, he should not trouble his hearers with the *Theory of Terrestrial Comets,* advocated by some ingenious and distinguished persons; nor with an examination of the *Lunar hypothesis,* supported as it was by famous and honourable names. He then examined other impregnations of the atmosphere by dust of various kinds, odours, and effluvia, of numberless variety, together with miasmata and poisons; all concurring to constitute a mass or volume of vapours, concerning which, although we know a great deal, we have much still to learn.

These interpretations of the constitution of the atmosphere, led to an examination of its operation upon the health of animals, in producing sporadic, endemic, epidemic, and epizootic diseases; in the course of which, he spoke of the Tract of *Hippocrates on air, water and situation,* with strong commendation; and treated with becoming severity the quarantine systems of the Christian nations, in ascribing to Foreign nations through a pretended contagion, the evils that arose from filthiness and bad management on board their own vessels. He gave his opinion solemnly, that *if quarantines were abolished, and ship-cleaning substituted in their stead,* the alteration would infinitely redound to the credit of science, the honour of police, and the advantage of commerce. Prof. M. stated also his belief, that the *metallic nature* of the atmosphere, would solve many of the phenomena of electricity and lightning, more satisfactorily than had been done heretofore; and opened new views of the subject by comparing the grand metallic apparatus of the atmosphere, both as to exciting and conducting power, with the amalgam and wires of the electrical machine, and with the plates and troughs of the galvanic battery.

